# Purification and preliminary characterization of a xylanase from *Thermomyces lanuginosus* strain SS-8

**DOI:** 10.1007/s13205-011-0032-6

**Published:** 2011-10-22

**Authors:** Smriti Shrivastava, Pratyoosh Shukla, Kunal Mukhopadhyay

**Affiliations:** Enzyme Technology Laboratory, Department of Biotechnology, Birla Institute of Technology (Deemed University), Mesra, Ranchi, Jharkhand India

**Keywords:** Bioethanol, *Thermomyces lanuginosus*, Thermophilic fungi, Xylanase, Xylose

## Abstract

*Thermomyces lanuginosus* SS-8 was isolated from soil samples that had been collected from near self-heating plant material and its extracellular cellulase-free xylanase purified approximately 160-fold using ion exchange chromatography and continuous elution electrophoresis. This xylanase was thermoactive (optimum temperature 60 °C) at pH 6.0 and had a molecular weight of 23.79 kDa as indicated by SDS-PAGE electrophoresis. The xylanase rapidly hydrolyzed xylan directly to xylose without the production of intermediary xylo-oligosaccharides within 15 min of incubation under optimum conditions. This trait of rapidly degrading xylan to xylose as a sole end-product could have biotechnological potential in degradation of agro-wastes for bioethanol manufacturing industry.

Xylan is the second most abundant heteropolymer in nature (Biely et al. 1985) and is found in close association with cellulose and lignin in plant cell wall. Xylan is formed by a backbone of β-1,4-d-xylopyranosyl residues, and different substitute groups exist in the side chain. Hence, for complete degradation of these polysaccharides an enzymatic complex comprising xylanases (β-1,4-d-xylan xylanohydrolase, EC 3.2.1.8) and β-xylosidases (β-1,4-d-xylan xylohydrolase, EC 3.2.1.37) are required. Xylanases cleave internal xylosidic linkages producing xylo-oligosaccharides, while β-xylosidases hydrolyze the oligosaccharides releasing xylose (Sunna and Antranikian 1997). Xylotriose has been reported to be the smallest xylo-oligosaccharide released during hydrolysis of xylan by xylanases (Ryan et al. 2003). As resistance to thermal inactivation in many industrial applications has become a desirable property of enzymes Singh et al. 2000), there has been an increasing focus on thermostable xylanases. Most of the current applications of thermostable xylanases are in pulp and paper industries, in which these enzymes are active at high temperature and alkaline pH (Beg et al. 2001). Thermostable xylanases have potential applications in fruit and vegetable processing, brewing, wine production, baking, animal feeds, starch, textiles industries, etc. (Beg et al. 2001; Viikari et al. 1994; Subramaniyan and Prema 2002). In addition, xylanases have also been found to play important roles in plant tissues in fruit softening, seed germination and plant defense systems (Prade 1995; Deising and Mendgen 1991). Cellulase-free xylanases have applications in textile industries. Several strains of the Ascomycetes fungi, *Thermomyces lanuginosus* (formerly known as *Humicola lanuginose*) are reported to produce high levels of cellulase-free thermostable β-xylanases belonging to family 11 of glycosyl hydrolases (Singh et al. 2003; Gaffney et al. 2009; Shrivastava et al. 2009). In the present study a thermophilic fungal strain SS-8 was identified as *T. lanuginosus* based on the sequencing of its GenBank accession number: GQ469970. Strain SS-8 was found to produce a novel extracellular xylanase whose characteristics are communicated here.

Soil samples adjacent to self-heating plant debris and wastes were collected from various locations in Jharkhand State, India. Fungi were isolated and screened for extracellular xylanase activity on Potato Dextrose Agar (PDA) plates containing 0.15% Remazol brilliant blue xylan (RBB-xylan, Sigma-Aldrich Chemei GmbH, Steinheim, Germany). Colonies showing clear zone of degradation of RBB-xylan were isolated and screened to select and prioritize the highest xylanase producers. For this the fungal isolates were grown on a production medium. Production medium (pH 6.5) contained yeast extract (Himedia, India) 1.5%, KH_2_PO_4_ (Himedia, India) 0.5% and 1.5% wheat bran (obtained from a local farmhouse). The fungal isolates were inoculated into production medium followed by incubation at 50 °C for 5 days using submerged fermentation. Xylanase activity was assayed using 2% (w/v) OSX (Oat Spelts Xylan) in 50 mM sodium acetate buffer, pH 5.3 at 50 °C (Biely et al. 1985). The reducing sugars liberated were quantified by the dinitrosalicylic acid (DNS) method (Miller 1959) using xylose (SigmaUltra, Sigma-Aldrich, St. Louis, MO, USA) as the standard. The amount of reducing sugars present was measured at 540 nm in a UV–Vis spectrophotometer (PerkinElmer Life and Analytical Sciences, Inc, Waltham, MA, USA). One unit of xylanase activity is defined as the amount of enzyme required to release 1 μmol of reducing sugar equivalent to xylose per min at 50 °C, pH 5.3. Twenty-five fungal strains were isolated from the soil samples of which 11 were extracellular xylanase producers. Strain SS-8 was observed to be the highest xylanase producer among all the 11 isolates and was therefore selected for further studies.

The morphology of strain SS-8 was determined by staining with lactophenol blue (Leck 1999) and calcoflour (Hageage and Harrington 1984). The cells were filamentous with septate hyphae, white in color, which simultaneously turned to yellow and then brown as they matured. They possessed aleurioconidia with a diameter range of 0.75–11 μm depending on stage of maturity. The 18S rDNA Internal Transcribed Spacer (ITS) region was sequenced commercially by Bangalore Genei, Bangalore, India. The nucleotide sequence (LENGTH) was analyzed using the BLASTn search program (http://blast.ncbi.nlm.nih.gov/) against the Genbank nucleotide (nr/nt) collection. BLAST analysis suggested that strain SS-8 was a strain of *T. lanuginosus*.

Strain SS-8 was cultured in production medium as described above and the xylanase was purified. For this, after 5 days incubation, the broth was clarified by centrifugation and the supernatant filtered through a 0.45 μm hydrophilic polyamide (HNWP) nylon membrane filter (Millipore Ireland B.V., Carrigtwohill, Ireland) using a vacuum manifold. The filtrate was purified using an anion exchange column (HiTrap Q FF 5 ml, GE Healthcare Bio-Sciences AB, Uppsala, Sweden) fitted to a low pressure chromatography system (ÄKTA Prime, GE Healthcare Bio-Sciences AB, Uppsala, Sweden) that had been previously equilibrated with 25 mM Tris–HCl buffer (pH 7.5). The analyte was eluted in 2.5-ml fractions with a linear gradient of 0–1 M NaCl in 25 MM Tris–HCl buffer (pH 7.5) at a flow rate 1 ml min^−1^). Fractions containing xylanase were identified using xylanase assays, pooled and subjected to continuous elution electrophoresis (CEE). For this, a Mini Prep Cell (Bio-Rad Laboratories, Hercules, CA, USA) containing 15% native polyacrylamide gel was used. An Econo System low pressure chromatography components, including peristaltic pump and fraction collector (Bio-Rad Laboratories, Hercules, CA, USA) were used for elution of the protein fractions with power supplied at 300 V and flow rate of 100 μl min^−1^. The elute was passed through an Amicon 10000 MOCO Ultra 50 centrifugal filter (Millipore Corporation, Bedford, MA) to remove contaminants and concentrate the enzyme. Samples were removed during purification and assayed for xylanase and cellulase activities and for protein concentration. Protein concentration was determined by the method of Bradford (1976) using bovine serum albumin as standard. Cellulase activity was assayed as described for xylanase assay except that carboxy methyl cellulose (CMC) was substituted instead of xylan. Specific activity is expressed as unit per milligram of protein. Molecular weight of the purified xylanase was determined using SDS-PAGE gel electrophoresis followed by staining the gel with Deep Purple (GE Healthcare Bio-Sciences Ltd., Buckinghamshire, England) as recommended by the manufacturer. Low molecular weight protein markers (PMW-L) from Bangalore Genei, Bangalore, India, were used as standards. The gel was scanned at 532 nm on a Typhoon 9410 multimode image analyzer (GE Healthcare, Sunnyvale, CA, USA) and the molecular weight was determined using ImageQuant TL 1D gel analysis software v2003.3 (GE Healthcare Bio-Sciences Ltd., Buckinghamshire, England) against the protein markers.

Data on the purification of the xylanase at various steps is summarized in Table 1. Anion exchange chromatography using HiTrap Q showed a single peak with xylanase activity (Fig. 1). The native PAGE on Mini Prep Cell (peak A, Fig. 2) shows the enzyme purified to homogeneity. A 166-fold purification was achieved from the culture filtrate. The xylanase was found to be cellulase-free at all stages of purification. A single protein band was observed on a 18% SDS-PAGE, suggesting that the xylanase was made of a single polypeptide chain with a molecular mass of 23.79 kDa as estimated by using the ImageQuant 1D gel analysis software (Fig. 3). The optimum temperature for the enzyme at pH 6.0 was determined to be 60 °C (Fig. 4**).**

**Table 1:** Summary of purification steps of xylanase from *T. lanuginosus* SS-8 grown on wheat bran as substrate

Purification steps	Total proteins (mg)	Total activity (U)	Specific activity (U mg^−1^)	Purification (fold)	Yield (%)
Crude culture filtrate	1,544.4	6,800	4.40	1	100
Ultrafiltration retentate	34.18	1,382.4	40.44	9.2	20.3
HiTrap Q FF	3.37	338.4	100.45	22.8	4.98
CEE	0.15	109.3	728.73	165.5	1.61

*CEE* continuous elution electrophoresis

**Fig. 1:**
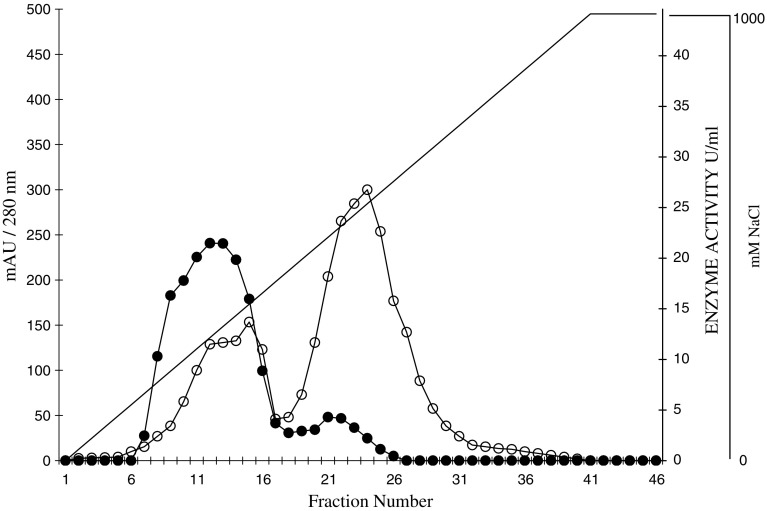
Elution profile of anion exchange chromatography of xylanase on HiTrap Q FF. Xylanase activity (*filled circles*); total protein (*empty circles* absorbance at 280 nm; NaCl (*straight line*)

**Fig. 2:**
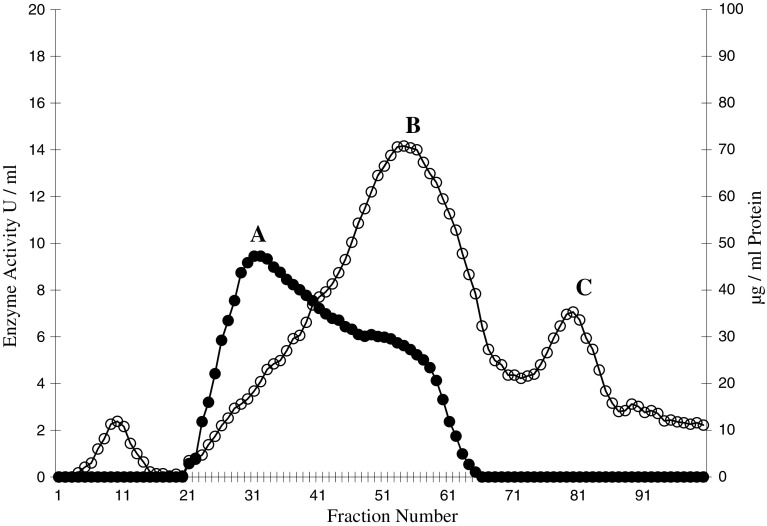
Elution profile of continuous elution electrophoresis for purification of xylanase on native polyacrylamide gel (15%). Peak *A* represents purified xylanase. Xylanase activity (*filled circle*) and total protein (*empty circle* absorbance at 280 nm)

**Fig. 3:**
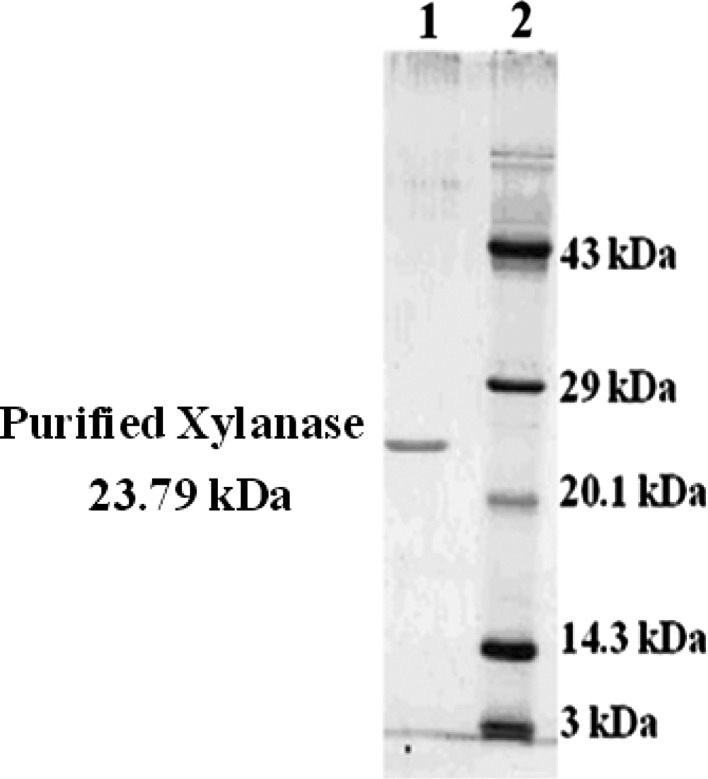
Molecular weight of purified xylanase from *T. lanuginosus* SS-8. SDS-PAGE (18%)*: lane 1* purified xylanase, *lane 2* PMW-L marker

**Fig. 4:**
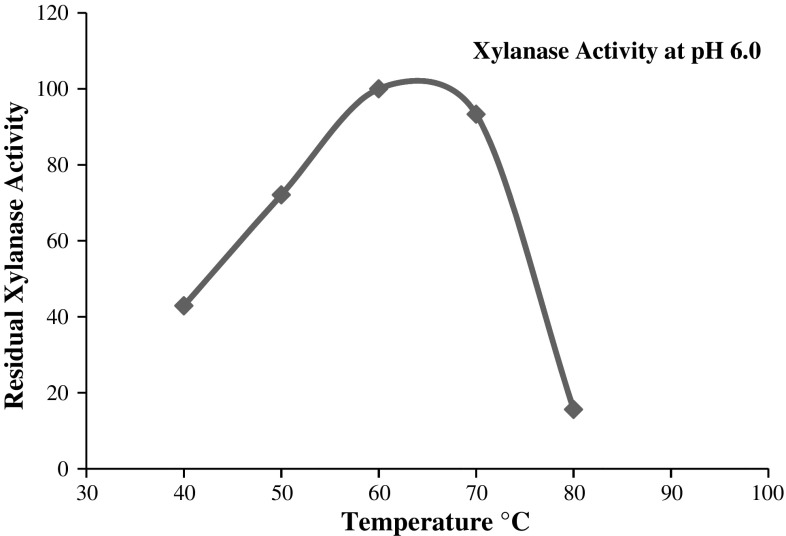
Optimum temperature of activity of xylanase from strain SS-8 at pH 6.0

The end-products of xylanase hydrolysis were determined by thin layer chromatography (TLC). For this, 3 ml of the xylan solution (10 mg OSX in 5.0 ml 50 mM sodium acetate buffer, pH 6.0) was incubated with 8 Units of purified enzyme at 60 °C. 0.3-ml aliquots were retrieved at intervals ranging from 0.25 to 72 h. Assay reactions without added enzyme were used as controls. 2 μl were spotted on Silica gel/TLC-cards, DC-Alufolien-Kieselgel (Fluka, Sigma-Aldrich, Seelz, Germany) and the hydrolysis products analyzed according to the method of Jiang et al. (2004). Aliquots of 0.3 ml were taken after 0.25, 0.5, 1, 3, 6, 12, 24, 48 and 72 h of incubation and concentrated to 0.2 ml. Blanks (1 and 72 h) containing xylan in buffer without xylanase were also incubated at 60 °C. The end-products of xylan degradation by the purified xylanase from incubations at different time intervals always showed the presence of xylose and no xylo-oligosaccharide intermediates were detected. Most xylanases reported to date (Ryan et al. 2003; Li et al. 2005; Sapre et al. 2005; Khandeparkar and Bhosle 2006; Li et al. 2006; Knob and Carmona 2009; Jiang et al. 2010; Hung et al. 2011) produce various xylo-oligosaccharides upon hydrolysis of xylan which are further hydrolyzed to xylose by xylosidases (Whistler and Masek 1955; Kulkarni et al. 1999). Even multifunctional xylanases (Ashabil and Burhan 2009; Xue et al. 2009) containing different combinations of xylan-degrading enzymes are unable to directly produce xylose as the end-product. This makes the xylanase from *T. lanuginosus* SS-8 unique amongst xylanases. The purified xylanase was also found to be fast acting as it completely hydrolyzed xylan to xylose within 15 min of incubation at 60 °C (Fig. 5). The fast acting, xylose producing, thermostable xylanase from *T. lanuginosus* SS-8 may have potential in degrading xylan-rich agro-wastes to ethanol and further research is currently underway in our laboratory.

**Fig. 5:**
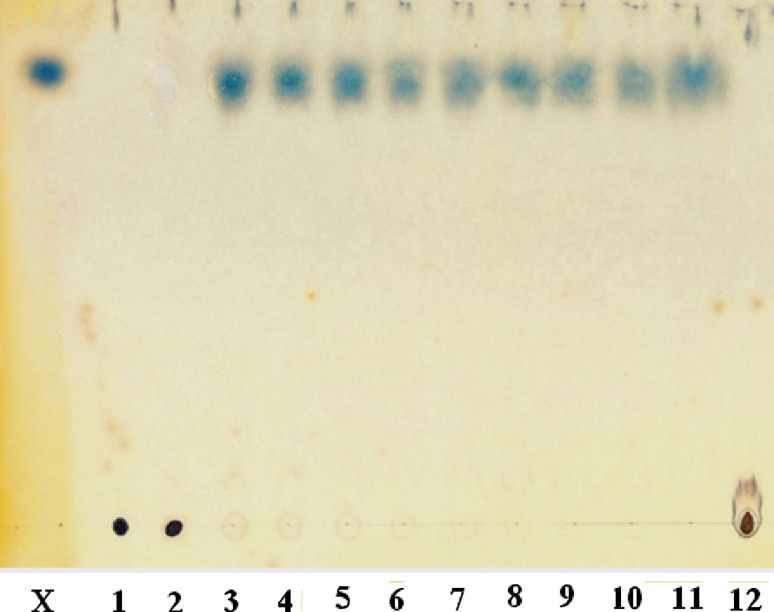
Analysis of hydrolysis product of oat spelts xylan degraded by xylanase from *T. lanuginosus* SS-8. *Lane X* xylose standard, *lane 1* xylan 0 h blank, *lane 2* 0 h hydrolysis, *lane 3* 0.25 h hydrolysis, *lane 4* 0.5 h hydrolysis, *lane 5* 1 h hydrolysis, *lane 6* 3 h hydrolysis, *lane 7* 6 h hydrolysis, *lane 8* 12 h hydrolysis, *lane 9* 24 h hydrolysis, *lane 10* 48 h hydrolysis, *lane 11* 72 h hydrolysis, *lane 12* xylan 1 h blank
